# The Risk of Immune-Related Thyroid Dysfunction Induced by PD-1/PD-L1 Inhibitors in Cancer Patients: An Updated Systematic Review and Meta-Analysis

**DOI:** 10.3389/fonc.2021.667650

**Published:** 2021-07-12

**Authors:** Yuan Tian, Ran Li, Yan Liu, Meng Li, Yuxiao Song, Yan Zheng, Aiqin Gao, Qing Wen, Guohai Su, Yuping Sun

**Affiliations:** ^1^ Department of Oncology, Jinan Central Hospital Affiliated to Shandong University, Jinan, China; ^2^ Special Care Department of Proton Therapy Center, Shandong Cancer Hospital and Institute, Shandong First Medical University and Shandong Academy of Medical Sciences, Jinan, China; ^3^ Department of Oncology, Jinan Central Hospital, Weifang Medical University, Weifang, China; ^4^ Qilu Hospital of Shandong University, Jinan, China; ^5^ Human Resources Department, Jinan Central Hospital Affiliated to Shandong University, Jinan, China; ^6^ Jinan Center for Disease Control and Prevention, Jinan, China; ^7^ Research Center of Translational Medicine, Jinan Central Hospital Affiliated to Shandong First Medical University, Jinan, China; ^8^ Research Center of Translational Medicine, Jinan Central Hospital, Cheeloo College of Medicine, Shandong University, Jinan, China; ^9^ Jinan Clinical Research Center of Shandong First Medical University, Jinan, China; ^10^ Department of Cardiovascular Diseases, Jinan Central Hospital Affiliated to Shandong University, Jinan, China; ^11^ Department of Oncology, Jinan Central Hospital Affiliated to Shandong First Medical University, Jinan, China

**Keywords:** thyroid dysfunction, PD-1/PD-L1 inhibitors, cancer, meta-analysis, risk

## Abstract

**Background:**

Thyroid dysfunction is common for cancer patients receiving PD-1/PD-L1 inhibitor therapies. To clarify the incidence risk of thyroid dysfunction would be important for guiding anti-PD-1 and anti-PD-L1 immunotherapy. Therefore, the updated meta-analysis was conducted to evaluate the incidence risk of thyroid dysfunction caused by PD-1/PD-L1 inhibitors.

**Methods:**

PD-1/PD-L1 inhibitor related clinical trials were collected by a systematic search of the PubMed. Some relevant studies were identified by a manual search. The incidence risk of all grades and grades 3-5 was analyzed and evaluated by random effect model. The Newcastle Ottawa Scale was used for the quality assessment of all clinical trials.

**Results:**

Forty-three clinical trials were collected. Compared with chemotherapy, the risk of hypothyroidism of all grades was significantly higher (OR=7.15, 95%CI:[4.85, 10.55], I^2^ = 40%, Z=9.91(*P <*0.00001)) in PD-1/PD-L1 group. Similar results could also be noted, when the control group was placebo or CTLA-4. When PD-1/PD-L1 was combined with other treatments for cancer patients, the risk of hypothyroidism of all grades was also significantly increased. Similar to the analysis results of hypothyroidism, PD-1/PD-L1 inhibitors played the same role in increasing the risk of hyperthyroidism and thyroiditis. Few significant analysis results was noted, when the risk of thyroid dysfunction of grades 3-5 was assessed.

**Conclusion:**

Whether used alone or in combination with other anti-tumor drugs, PD-1/PD-L1 inhibitors increased the risk of thyroid dysfunction, especially for hypothyroidism. Furthermore, PD-1/PD-L1 was better than chemotherapy and CTLA-4 in increasing the risk of thyroid dysfunction.

## Introduction

Programmed cell death protein 1 (PD-1) and its ligand (PD-L1) inhibitors, developed to overcome the immune escape mechanisms of cancer progression and manipulate the immune system to recognize and attack cancer cells, have been widely used for cancers (1). While achieving satisfactory clinical anti-tumor treatment effects, more and more drug-induced toxic and side effects have also been reported, and more and more attention has been drawn from clinicians (1–3). Treatment guidelines for PD-1/PD-L1 related side effects have been made and used to guide clinical works (2).

Thyroid dysfunction was one of the common toxic side effects of PD-1/PD-L1 inhibitors and had been reported in plenty of clinical trials (4–50). Moreover, It was reported that the incidence of PD-1/PD-L1 induced thyroid dysfunction was related to the clinical response and the prognosis of patients (51, 52). Therefore, clarifying the incidence risk of PD-1/PD-L1 related thyroid dysfunction would be of great significance for guiding clinical immunotherapy and judging the prognosis (51, 52). Although thyroid dysfunction might appear in different forms (53), hyperthyroidism, hypothyroidism, and thyroiditis were still the most common manifestations (1), which were also reported most frequently in clinical trials (4–50). Due to more and more clinical trials investigating the clinical efficacy and safety of PD-1/PD-L1 in cancer patients have been finished in recent two years (4–23), we conducted this updated meta-analysis to reassess the incidence risk of PD-1/PD-L1 induced hyperthyroidism, hypothyroidism, and thyroiditis.

## Method

The process of the meta-analysis was put into practice followed the guidelines of the Preferred Reporting Items for Systematic Reviews and Meta-analyses (PRISMA) (54).

### Types of Enrolled Studies

Clinical trials, involving PD-1 or PD-L1 inhibitors, were identified by the PubMed search. Hematological malignancies were excluded first. Phase III clinical trials for all kinds of cancer patients would be taken as the priority. Clinical trials, reported with partial results or belonging to other phases, would be arranged in an alternative location. For all clinical trials included in the study, the control group was necessary, but there was no specific requirement for the treatment regimen of them. The results of the enrolled clinical trial must be reported in English.

### Search Strategy

Just as proposed by the PRISMA, keywords (neoplasm, cancer, precancer, malignant, premalignant, tumor, PD-1, PD-L1, and clinical trial) for search were set according to the PICOS (participants, interventions, comparisons, outcomes, and study design) guidelines (54). The range of published time was set between Nov 23, 2010 and Nov 23, 2020. Four members of us were appointed for eligibility assessment and data extraction. In the case of duplicated reports of the same clinical trial, only one of them was used for the final analysis, and others would be included in the systematic review. The corresponding authors (Yuping Sun and Guohai Su) had the right to deal with all results and disagreements.

### Evaluation of Study Quality and Publication Bias

Assessment for publication bias and risk of bias of individual trials were finished by Funnel plots, Egger’s test, Harbord’s test, and the Newcastle-Ottawa scale (NOS) (54–59). Risk of bias summary, including selection bias, performance bias, detection bias, attrition bias, reporting bias and other bias, would be checked and shown in a single figure. A *P-value* of <0.05 was used as the cut-off value for statistical significance.

### Outcome and Exposure of Interest

Baseline characteristics of all enrolled clinical trials, including duplicating reported ones, would be collected and summarized in a table. Grading of thyroid dysfunction, including hyperthyroidism, hypothyroidism, and thyroiditis, ranging from 1 (mild symptoms that do not interfere with activities of daily living) to 5 (fatal thyroid toxicities), was collected and gathered in excel tables. Dichotomous data would be given a priority, and other types of data would be collected first and then converted into dichotomous data.

### Assessment of Heterogeneity and Statistical Analysis

Heterogeneity of all the data, identified by Cochrane’s Q statistic test, was assessed by the DerSimonian-Laird method and quantified by I^2^ values (54, 59). Three different grades, including low, moderate, and high, were divided according to I^2^ values ( < 25%, 25-50%, and > 50%). All the process of analyses was finished by the software Review Manager 5.3. The random effect model (RE) was used to deal with all the data to calculate odds ratio (OR) and their corresponding 95% confidence interval (CI) (60). The fixed effects (FE) model was only used for calculation of the funnel plots. All reported *P* values are 2-sided, and *P*<0.05 was taken to indicate statistically significance. Subgroup and stratification analyses would be performed according to tumor types, treatment regimens, and PD-1/PD-L1 inhibitors.

## Results

### Literature Search Results

**Figure 1:**
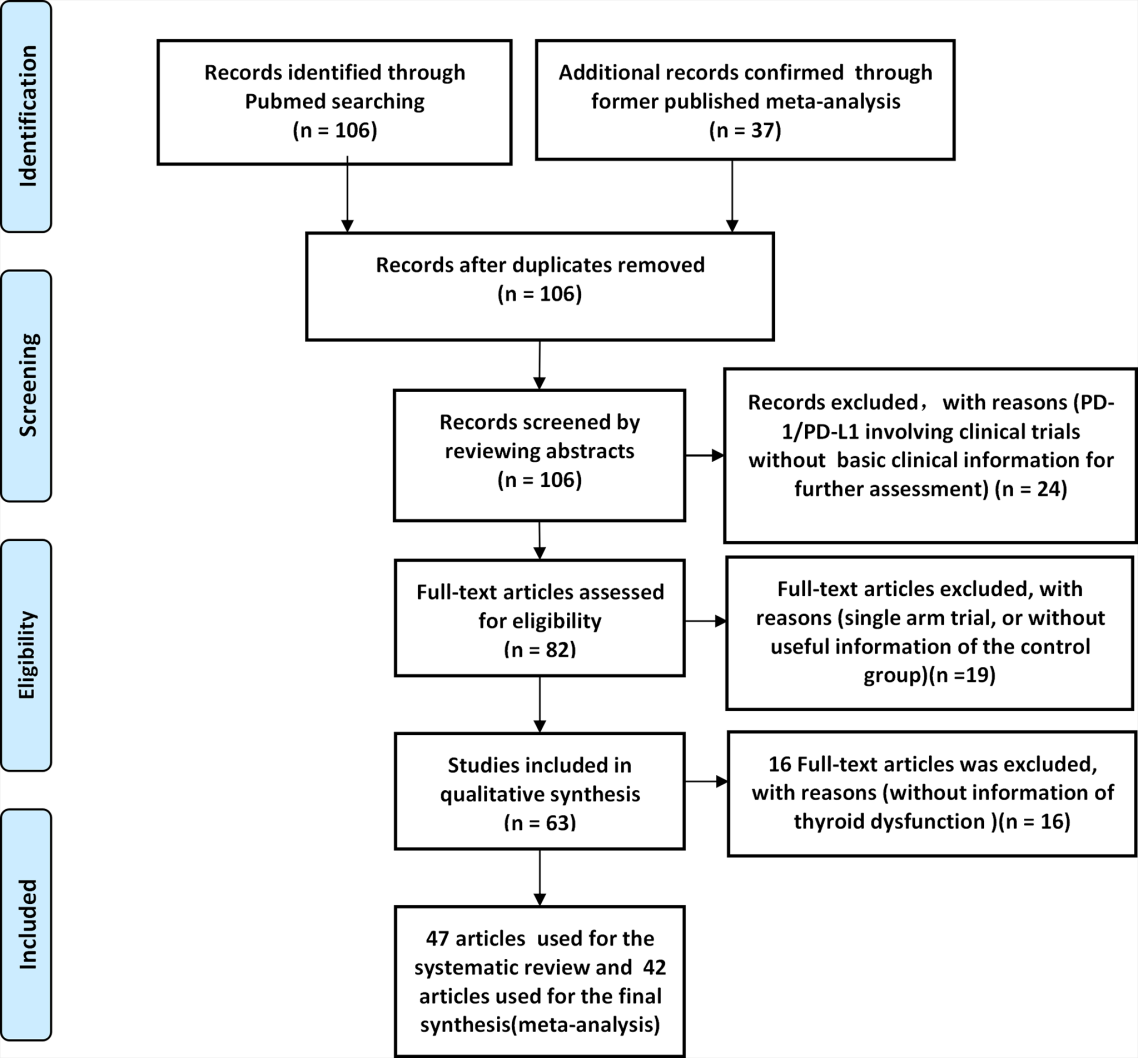
The PRISMA flow diagram of the screening process.

The PRISMA flow diagram was shown in (**Figure 1**), while the bias assessment summary of all enrolled clinical trials were provided in (**Supplementary Figure 1**). A total of 589 published studies was found by PubMed search, while 37 studies were gotten from the former published meta-analysis (61–63). After eligibility assessment, 5 articles were only used for the systematic review (13, 20–23), while 42 articles were used for the final comprehensive analysis (4–12, 14–19, 24–50). The clinical trial ‘CheckMate 067’ (NCT01844505) was reported 4 times (47–50), while the clinical trial ‘PACIFIC’ (NCT02125461) was reported 2 times (45, 46).

### Characteristics of Identified Trials

Forty-three clinical trials, including 1 phase I (20), 1 phase I/II (40), 3 phase II (6, 9, 41), 1 phase II/III (39), and 37 phase III (4, 5, 7, 8, 10–12, 14–19, 21–38, 42–50), were collected and listed in (**Table 1**). Among all of them, 25 clinical trials (involving 28 articles) was found to be PD-1 related (4, 6, 7, 11, 12, 15, 16, 23, 25, 27–29, 32, 34–44, 47–50), while 18 clinical trials (involving 19 articles) was reported to be PD-L1 related (5, 8–13, 16, 17, 20–22, 24, 26, 30, 31, 33, 45, 46). PD-1 or PD-L1 inhibitors were prescribed as the first line treatment regimen in 22 clinical trials (7, 8, 10–12, 14, 16, 18, 20–23, 27, 29, 33, 36, 37, 41, 47–50), and previous therapy was found in the other 21 clinical trials (4–6, 9, 13, 15, 17, 19, 24–26, 28, 34, 35, 38–40, 42–46). In all the clinical trials included in the study, 8 tumor types are mainly involved, of which lung cancer accounts for the largest proportion (**Table 1**) (12–14, 16, 17, 24, 26, 27, 29, 30, 32, 33, 37, 39, 40, 42, 44–46).

**Table 1:** Baseline characteristics of all enrolled clinical trials (N = 47 articles of 43 clinical trials).

NO	Reference	NCT number	Drug Name	Treatment Regimen	Previous therapy	Phase	Involving Patients	Hypothyr-oidism	Hyperthyroidism	Thyroiditis	Tumor Type
1	Huang et al. (4)	NCT03099382 (ESCORT)	Camrelizumab (PD-1)	Camrelizumab VS. Docetaxel	YES	III	448	41	N/A	N/A	OSCC
2	Powles et al. (5)	NCT02302807 (IMvigor211)	Avelumab (PD-L1)	Avelumab VS. Placebo	YES	III	689	42	21	N/A	UC
3	Zimmer et al. (6)	NCT02523313 (IMMUNED)	Nivolumab (PD-1)	Nivolumab VS. (Nivolumab + Ipilimumab)/Placebo	YES	II	162	16	25	4	Melanoma
4	Schmid et al. (7)	NCT03036488 (KEYNOTE-522)	Pembrolizumab (PD-1)	(Pembrolizumab + (DC/EC)) VS. (Placebo + (DC/EC))	NO	III	1170	120	40	16	TNBC
5	Mittendorf et al. (8)	NCT03197935 (IMpassion031)	Atezolizumab (PD-L1)	(Atezolizumab + nPDC) VS. (Placebo + nPDC)	NO	III	331	13	5	N/A	TNBC
6	Emens et al. (9)	NCT02924883 (KATE2)	Atezolizumab (PD-L1)	(Atezolizumab + TE) VS. (Placebo + TE )	YES	II	200	N/A	2	N/A	BC
7	Gutzmer et al. (10)	NCT02908672 (IMspire150)	Atezolizumab (PD-L1)	(Atezolizumab + VC) VS. (Placebo + VC)	NO	III	511	55	60	N/A	Melanoma
8	Galsky et al. (11)	NCT02807636 (IMvigor130)	Atezolizumab (PD-L1)	(Atezolizumab + Chemotherapy) VS. (Atezolizumab/ Chemotherapy)	NO	III	807	99	55	N/A	UC
9	Herbst et al. (12)	NCT02409342 (IMpower110)	Atezolizumab (PD-L1)	Atezolizumab VS. Chemotherapy (Platinum-based)	NO	III	549	31	15	N/A	NSCLC
10	Reck et al. (13)	NCT02366143 (IMpower150)	Atezolizumab (PD-L1)	ACP VS. ABCP	YES	III	793	90	27	N/A	NSCLC
11	Mok et al. (14)	NCT02220894 (KEYNOTE-042)	Pembrolizumab (PD-1)	Pembrolizumab VS. Chemotherapy(platinum-based)	NO	III	1251	86	43	10	NSCLC
12	Cohen et al. (15)	NCT02252042 (KEYNOTE-040)	Pembrolizumab (PD-1)	Pembrolizumab VS. (Methotrexate,Docetaxel/ Cetuximab)	YES	III	480	46	6	N/A	HNSCC
13	Paz-Ares et al. (16)	NCT03043872 (CASPIAN)	Durvalumab (PD-L1)	(Durvalumab + EP) VS. EP	NO	III	531	23	22	4	SCLC
14	West et al. (17)	NCT02367781 (IMpower130)	Atezolizumab (PD-L1)	(Atezolizumab + CnP) VS. CnP	YES	III	705	71	24	N/A	NSCLC
15	Burtness et al. (18)	NCT02358031 (KEYNOTE-048)	Pembrolizumab (PD-1)	Pembrolizumab VS. (Pembrolizumab + Chemotherapy)/ (Cetuximab + Chemotherapy)	NO	III	863	107	23	N/A	HNSCC
16	Kato et al. (19)	NCT02569242 (ATTRACTION-3)	Nivolumab (PD-1)	Nivolumab VS. Paclitaxel/Docetaxel	YES	III	417	2	N/A	N/A	OSCC
17	Sullivan et al. (20)	NCT01656642	Atezolizumab (PD-L1)	(Atezolizumab + vemurafenib) VS. (Atezolizumab + Cobimetinib + Vemurafenib)	NO	I	56	10	N/A	N/A	Melanoma
18	Rini et al. (21)	NCT02420821 (IMmotion151)	Atezolizumab (PD-L1)	(Atezolizumab + Bevacizumab) VS. Sunitinib	NO	III	897	215	46	N/A	RCC
19	Motzer (22)	NCT02684006 (JAVELIN Renal 101)	Avelumab (PD-L1)	(Avelumab + Axitinib) VS. Sunitinib	NO	III	873	169	N/A	N/A	RCC
20	Motzer et al. (23)	NCT02231749 (CheckMate 214)	Nivolumab (PD-1)	(Nivolumab + Ipilimumab) VS. Sunitinib	NO	III	1082	228	72	16	RCC
21	Barlesi et al. (24)	NCT02395172 (JAVELIN Lung 200)	Avelumab (PD-L1)	Avelumab VS. Docetaxel	YES	III	758	22	5	3	NSCLC
22	Shitara et al. (25)	NCT02370498 (KEYNOTE-061)	Pembrolizumab (PD-1)	Pembrolizumab VS. Paclitaxel	YES	III	570	24	13	N/A	GGOJC
23	Hida et al. (26)	NCT02008227	Atezolizumab (PD-L1)	Atezolizumab VS. Docetaxel	YES	III	101	4	3	N/A	NSCLC
24	Gandhi et al. (27)	NCT02578680 (KEYNOTE-189)	Pembrolizumab (PD-1)	Pembrolizumab VS. Placebo	NO	III	607	32	22	1	NSCLC
25	Eggermont et al. (28)	NCT02362594	Pembrolizumab (PD-1)	Pembrolizumab VS. Placebo	YES	III	1011	87	58	17	Melanoma
26	Paz-Ares et al. (29)	NCT02775435 (KEYNOTE-407)	Pembrolizumab (PD-1)	Pembrolizumab VS. Placebo	NO	III	558	27	22	4	NSCLC
27	Socinski et al. (30)	NCT02366143 (IMpower150)	Atezolizumab (PD-L1)	(Atezolizumab + BCP) VS. BCP	NO	III	787	65	21	N/A	NSCLC
28	Schmid et al. (31)	NCT02425891 (IMpassion130)	Atezolizumab (PD-L1)	(Atezolizumab + Nab-Paclitaxel) VS. (Placebo +Nab-Paclitaxel)	NO	III	890	97	26	N/A	TNBC
29	Hellmann et al. (32)	NCT02477826 (CheckMate 227)	Nivolumab (PD-1)	Nivolumab VS. (Nivolumab + Ipilimumab)/Chemotherapy	NO	III	1537	92	N/A	N/A	NSCLC
30	Horn et al. (33)	NCT02763579 (IMpower133)	Atezolizumab (PD-L1)	Atezolizumab VS. Placebo	NO	III	394	26	16	N/A	NSCLC
31	Bellmunt et al. (34)	NCT02256436 (KEYNOTE-045)	Pembrolizumab (PD-1)	Pembrolizumab VS. (Platinum-based + Paclitaxel, Docetaxel, or Vinflunine)	YES	III	521	20	11	2	UC
32	Kang et al. (35)	NCT02267343 (ONO-4538-12, ATTRACTION-2)	Nivolumab (PD-1)	Nivolumab VS. Placebo	YES	III	491	11	2	1	GGOJC
33	Schachter et al. (36)	NCT01866319 (KEYNOTE-006)	Pembrolizumab (PD-1)	Pembrolizumab VS. Ipilimumab	NO	III	811	55	N/A	N/A	Melanoma
34	Reck et al. (37)	NCT02142738 (KEYNOTE-024)	Pembrolizumab (PD-1)	Pembrolizumab VS. Chemotherapy	NO	III	304	16	14	4	NSCLC
35	Ferris et al. (38)	NCT02105636 (CheckMate 141)	Nivolumab (PD-1)	Nivolumab VS. Chemotherapy	YES	III	347	10	2	2	HNSCC
36	Herbst et al. (39)	NCT01905657 (KEYNOTE-010)	Pembrolizumab (PD-1)	Pembrolizumab VS. Docetaxel	YES	II/III	991	57	35	2	NSCLC
37	Antonia et al. (40)	NCT01928394 (CheckMate 032)	Nivolumab (PD-1)	Nivolumab VS. (Nivolumab+Ipilimumab)	YES	I/II	213	14	12	N/A	SCLC
38	Hodi et al. (41)	NCT01927419 (CheckMate 069)	Nivolumab (PD-1)	Ipilimumab VS. (Nivolumab + Ipilimumab)	NO	II	140	22	N/A	2	Melanoma
39	Borghaei et al. (42)	NCT01673867 (CheckMate 057)	Nivolumab (PD-1)	Nivolumab VS. Docetaxel	YES	III	555	19	4	1	NSCLC
40	Weber et al. (43)	NCT01721746 (CheckMate 037)	Nivolumab (PD-1)	Nivolumab VS. (Dacarbazine/Paclitaxel + Carboplatin)	YES	III	370	15	6	N/A	Melanoma
41	Brahmer et al. (44)	NCT01642004 (CheckMate 017)	Nivolumab (PD-1)	Nivolumab VS. Docetaxel	YES	III	260	5	N/A	N/A	NSCLC
42	Antonia et al. (45)	NCT02125461 ( PACIFIC)	Durvalumab (PD-L1)	Durvalumab VS. Placebo	YES	III	709	59	36	N/A	NSCLC
43	Antonia et al. (46)										
44	Larkin et al. (47)	NCT01844505 (CheckMate 067)	Nivolumab (PD-1)	Nivolumab VS. (Nivolumab + Ipilimumab)/Ipilimumab	NO	III	937	100	52	17	Melanoma
45	Wolchok et al. (48)										
46	Hodi et al. (49)										
47	Larkin et al. (50)										

N/A, No Available; RCC, Renal Cell Carcinoma; NSCLC, Non Small Cell Lung Cancer; HNSCC, Head-and-Neck Squamous Cell Carcinoma; SCLC, Small Cell Lung Cancer; TNBC, Triple-Negative Breast Cancer; BC, Breast Cancer; UC, Urothelial Carcinoma; OSCC, Oesophageal Squamous Cell Carcinoma; HNSCC, Head-and-Neck Squamous Cell Carcinoma; RCC, Renal Cell Carcinoma; DC, Doxorubicin+Cyclophosphamide; EC, Epirubicin+Cyclophosphamide; GGOJC, Gastric or Gastro-Oesophageal Junction Cancer; CnP, Carboplatin+nab-paclitaxel; nPDC, nab-paclitaxel+ doxorubicin+cyclophosphamide; TE, Trastuzumab + Emtansine; VC, Vemurafenib + Cobimetinib; BCP, Bevacizumab+Carboplatin+Paclitaxel; ACP, Atezolizumab + Carboplatin + Paclitaxel; ABCP, Atezolizumab + Bevacizumab + Carboplatin + Paclitaxel.

### Risk of Bias

Bias assessment summary was provided in (**Supplementary Figure 1**). High attrition bias was only found in 1 articles (**Supplementary Figure 1**) (47), while unclear risk was identified in 21 articles (4, 8, 9, 13, 18–22, 25, 26, 30, 32, 36, 40, 41, 43–47). Publication bias assessment was displayed in the form of funnel plots, which were provided in the supplement (**Supplementary Figures 2**–**6**).

### Risk of Hypothyroidism

Hypothyroidism was identified in 42 clinical trials (4–8, 10–50), 36 of which were used for the final meta-analysis (4–8, 10–12, 14–19, 24–50). For high attrition bias, one reported results of CheckMate 067 was excluded (**Table 1**) (47).

**Figure 2:**
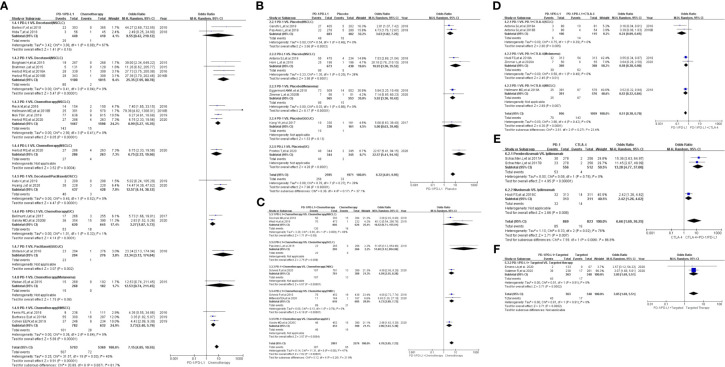
Forest plots of the risk of all-grade hypothyroidism. **(A)** The risk of hypothyroidism calculated by the random effect (RE) model (PD-1/PD-L1 VS. Chemotherapy): subgroup analysis was conducted based on PD-1/PD-L1, chemotherapy drugs and tumor types in both groups. **(B)** The risk of hypothyroidism calculated by the random effect (RE) model (PD-1/PD-L1 VS. Placebo): subgroup analysis was conducted based on PD-1/PD-L1 and tumor types in both groups. **(C)** The risk of hypothyroidism calculated by the random effect (RE) model (PD-1/PD-L1+Chemotherapy VS. Chemotherapy): subgroup analysis was conducted based on PD-1/PD-L1 and tumor types in both groups. **(D)** The risk of hypothyroidism calculated by the random effect (RE) model (PD-1/PD-L1 VS. PD-1/PD-L1+CTLA-4): subgroup analysis was conducted based on tumor types in the control group. **(E)** The risk of hypothyroidism calculated by the random effect (RE) model (PD-1 VS. CTLA-4): subgroup analysis was conducted based on the PD-1 group. **(F)** The risk of hypothyroidism calculated by the random effect (RE) model (PD-1/PD-L1+Targeted VS. Targeted).

**Figure 3:**
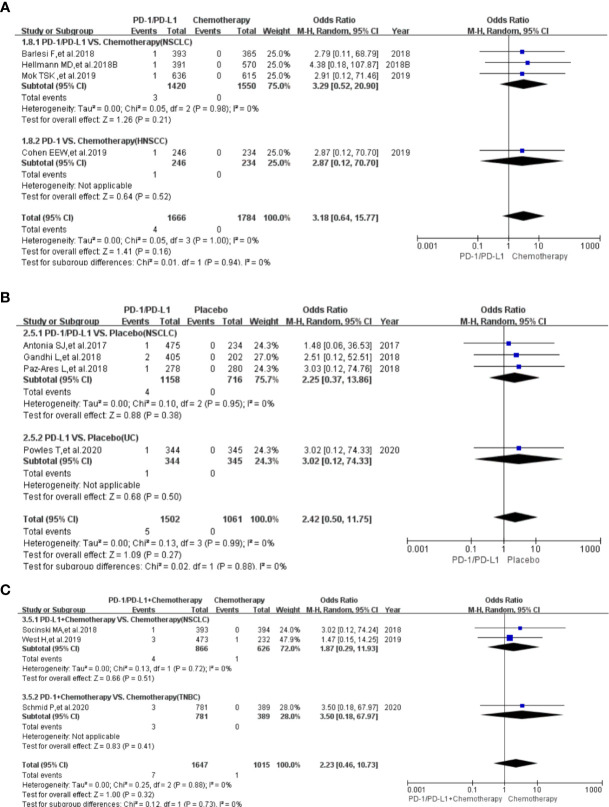
Forest plots of the risk of hypothyroidism for grades 3-5. **(A)** The risk of hypothyroidism calculated by the random effect (RE) model (PD-1/PD-L1 VS. Chemotherapy): subgroup analysis was conducted based on PD-1/PD-L1 and tumor types in both groups. **(B)** The risk of hypothyroidism calculated by the random effect (RE) model (PD-1/PD-L1 VS. Placebo): subgroup analysis was conducted based on PD-1/PD-L1 and tumor types in both groups. **(C)** The risk of hypothyroidism calculated by the random effect (RE) model (PD-1/PD-L1+Chemotherapy VS. Chemotherapy): subgroup analysis was conducted based on PD-1/PD-L1 and tumor types in both groups.

Compared with chemotherapy (PD-1/PD-L1 VS. Chemotherapy), the risk of hypothyroidism of all grades was significantly higher (OR=7.15, 95%CI:[4.85, 10.55], I^2^ = 40%, Z=9.91(*P <*0.00001); **Figure 2A**) (4, 11, 12, 14, 15, 18, 19, 24–26, 32, 34, 37–39, 42–44). Subgroup analysis suggested that PD-1 appeared to be associated with a higher incidence risk of hypothyroidism (OR=8.34, 95%CI:[5.24, 13.28], I^2^ = 37%, Z=8.94(*P <*0.00001); **Supplementary Figure 7**) (4, 14, 15, 18, 19, 25, 32, 34, 37–39, 42–44). Further stratification of subgroup analysis suggested that this risk trend was especially obvious in NSCLC subgroup (PD-1 VS. Docetaxel), when the control group was Docetaxel (OR=25.35, 95%CI:[7.95, 80.78], I^2^ = 0%, Z=5.47(*P <*0.00001)) (Chi^2^ = 20.89, df=8(*P*=0.007), I^2^ = 61.67%; **Figure 2A**) (39, 42, 44). Through subgroup analysis, moderate heterogeneity (I^2^ = 40%, **Figure 2A**) was considered to be mainly caused by one of NSCLC subgroups (PD-L1 VS. Docetaxel) (I^2^ = 67%, **Figure 2A**) (24, 26). No obvious publication bias was found in the funnel plot (**Supplementary Figure 2A**). No significant results was noted (OR=3.18, 95%CI:[0.64, 15.77], I^2^ = 0%, Z=1.41(*P =0.16*); **Figure 3A**), when the risk of hypothyroidism of grades 3-5 was assessed (14, 15, 24, 32). The corresponding funnel plot was shown in the supplement (**Supplementary Figure 3A**) (14, 15, 24, 32).

Compared with placebo (PD-1/PD-L1 VS. Placebo), the risk of hypothyroidism of all grades was significantly higher (OR=6.32, 95%CI:[4.01, 9.95], I^2^ = 20%, Z=7.96(*P <*0.00001); **Figure 2B**) (5, 6, 27–29, 33, 35, 46). Through subgroup analysis, low heterogeneity (I^2^ = 20%, **Figure 2B**) was considered to be mainly caused by one of NSCLC subgroups (PD-L1 VS. Chemotherapy) (I^2^ = 26%, **Figure 2B**) (33, 46). No obvious publication bias was found in the corresponding funnel plot (**Supplementary Figure 2B**). No significant results was noted (OR=2.42, 95%CI:[0.50, 11.75], I^2^ = 0%, Z=1.09(*P =0.27*); **Figure 3B**), when the risk of hypothyroidism of grades 3-5 was calculated (5, 27, 29, 45). The corresponding funnel plot was shown in the supplement (**Supplementary Figure 3B**) (5, 27, 29, 45).

When PD-1/PD-L1 combined with chemotherapy was compared with chemotherapy (PD-1/PD-L1+Chemotherapy VS. Chemotherapy), the risk of hypothyroidism of all grades was found to be significantly higher (OR=4.70, 95%CI:[3.05, 7.23], I^2^ = 47%, Z=7.02(*P <*0.00001); **Figure 2C**) in the PD-1/PD-L1 group (7, 8, 11, 16, 17, 30, 31). Through subgroup analysis, moderate heterogeneity (I^2^ = 47%, **Figure 2C**) was considered to be mainly caused by the NSCLC subgroup (I^2^ = 86%, **Figure 2C**) (17, 30). No obvious publication bias was found in the funnel plot (**Supplementary Figure 2C**).

No significant results was noted (OR=2.23, 95%CI:[0.46, 10.73], I^2^ = 0%, Z=1.00(*P =0.32*); **Figure 3C**), when the risk of hypothyroidism of grades 3-5 was assessed (7, 17, 30). The corresponding funnel plot was shown in the supplement (**Supplementary Figure 3C**) (7, 17, 30).

When PD-1/PD-L1 combined with CTLA-4 was compared with PD-1/PD-L1 (PD-1/PD-L1 VS. PD-1/PD-L1+CTLA-4), the risk of hypothyroidism of all grades was found to be significantly lower (OR=0.51, 95%CI:[0.38, 0.70], I^2^ = 0%, Z=4.30(*P <*0.00001); **Figure 2D**) in the PD-1/PD-L1 group (6, 32, 40, 49). No heterogeneity (I^2^ = 0%) was found. No obvious publication bias was found in the funnel plot (**Supplementary Figure 2D**). There were too few data to calculate the risk of hypothyroidism of grades 3-5 (49).

Compared with CTLA-4 (PD-1 VS. CTLA-4), the risk of hypothyroidism of all grades was found to be significantly higher (OR=6.66, 95%CI:[1.69, 26.25], I^2^ = 76%, Z=2.71(*P =0.007*); **Figure 2E**) in the PD-1 group (36, 49). Through subgroup analysis, high heterogeneity (I^2^ = 76%, **Figure 2E**) might be related to the Nivolumab subgroup (**Figure 2E**) (49). The corresponding funnel plot was shown in the supplement (**Supplementary Figure 3E**). No data of hypothyroidism of grades 3-5 was found.

When PD-1/PD-L1 combined with targeted therapy was compared with PD-1/PD-L1 (PD-1/PD-L1+Targeted VS. Targeted), the risk of hypothyroidism of all grades was found to be significantly increased (OR=3.05, 95%CI:[1.69, 5.51], I^2^ = 0%, Z=3.71(*P =0.0002*); **Figure 2F**) (9, 10). No heterogeneity (I^2^ = 0%) was found. No obvious publication bias was found in the funnel plot (**Supplementary Figure 2F**). No data of hypothyroidism of grades 3-5 was found.

### Risk of Hyperthyroidism

Hyperthyroidism was identified in 36 clinical trials (5–18, 21, 23–31, 33–35, 37–40, 42, 43, 45–50), 31 of which were used for the final meta-analysis (5–12, 14–18, 24–31, 33–35, 37–40, 42, 43, 45–50).

**Figure 4:**
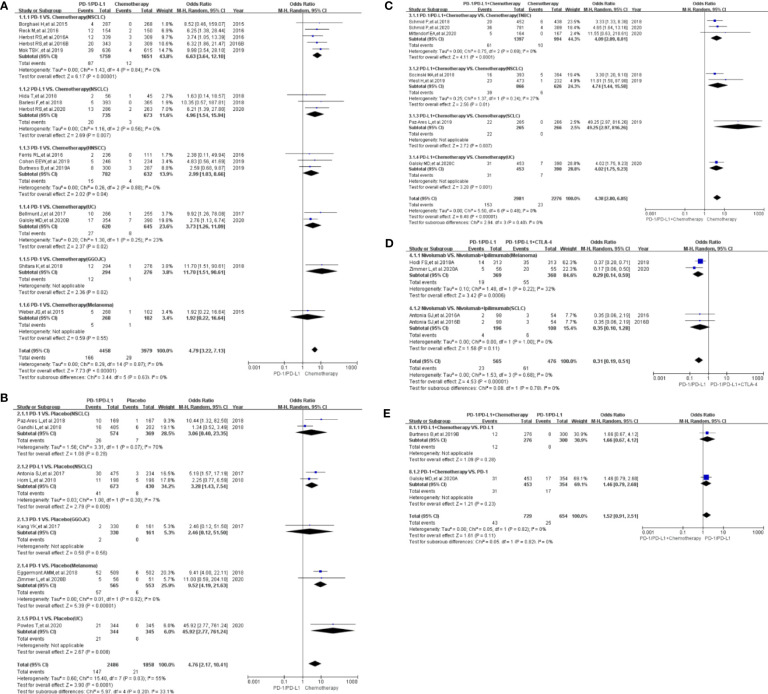
Forest plots of the risk of all-grade hyperthyroidism. **(A)** The risk of hyperthyroidism calculated by the random effect (RE) model (PD-1/PD-L1 VS. Chemotherapy): subgroup analysis was conducted based on PD-1/PD-L1 and tumor types in both groups. **(B)** The risk of hyperthyroidism calculated by the random effect (RE) model (PD-1/PD-L1 VS. Placebo): subgroup analysis was conducted based on PD-1/PD-L1 and tumor types in both groups. **(C)** The risk of hyperthyroidism calculated by the random effect (RE) model (PD-1/PD-L1+Chemotherapy VS. Chemotherapy): subgroup analysis was conducted based on PD-1/PD-L1 and tumor types in both groups. **(D)** The risk of hyperthyroidism calculated by the random effect (RE) model (PD-1/PD-L1 VS. PD-1/PD-L1+CTLA-4): subgroup analysis was conducted based on tumor types in the control group. **(E)** The risk of hyperthyroidism calculated by the random effect (RE) model (PD-1/PD-L1+chemotherapy VS. PD-1/PD-L1): subgroup analysis was conducted based on PD-1/PD-L1 in both groups.

**Figure 5:**
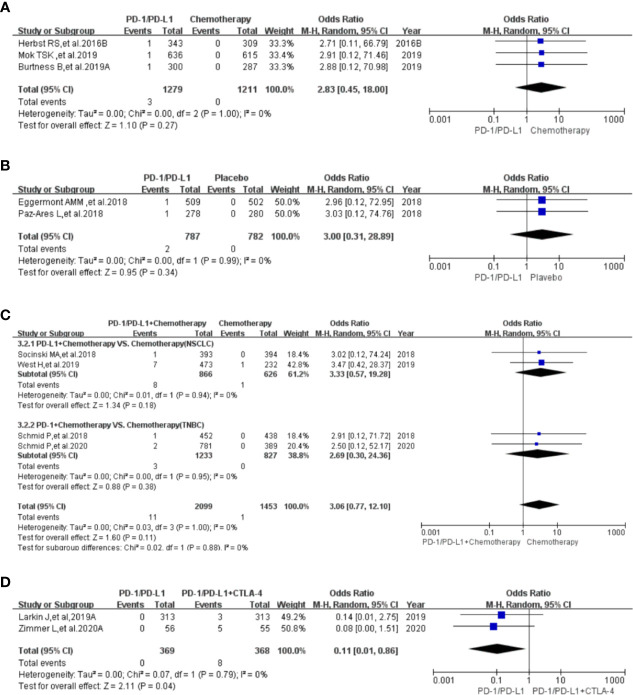
Forest plots of the risk of hyperthyroidism for grades 3-5. **(A)** The risk of hyperthyroidism calculated by the random effect (RE) model (PD-1/PD-L1 VS. Chemotherapy). **(B)** The risk of hyperthyroidism calculated by the random effect (RE) model (PD-1/PD-L1 VS. Placebo). **(C)** The risk of hyperthyroidism calculated by the random effect (RE) model (PD-1/PD-L1+Chemotherapy VS. Chemotherapy): subgroup analysis was conducted based on PD-1/PD-L1 and tumor types in both groups. **(D)** The risk of hyperthyroidism calculated by the random effect (RE) model (PD-1/PD-L1 VS. PD-1/PD-L1+CTLA-4).

Compared with chemotherapy (PD-1/PD-L1 *VS.* Chemotherapy), the risk of hyperthyroidism of all grades was significantly higher (OR=4.79, 95%CI:[3.22, 7.13], I^2^ = 0%, Z=7.73(*P <*0.00001); **Figure 4A**) in PD-1/PD-L1 group (11, 12, 14, 15, 18, 24–26, 34, 37–39, 42, 43). Subgroup analysis suggested that PD-1 appeared to be associated with a higher incidence risk of hyperthyroidism (OR=5.59, 95%CI:[3.46, 9.04], I^2^ = 0%, Z=7.03(*P <*0.00001); **Supplementary Figure 8**) (14, 15, 18, 25, 34, 37–39, 42, 43). However, no statistical significant difference was found between PD-1 and PD-L1 subgroup (*P =0.26*, **Supplementary Figure 8**). No heterogeneity (I^2^ = 0%) was found (**Figure 4A**). No obvious publication bias was found in the corresponding funnel plot (**Supplementary Figure 4A**). No significant results was noted (OR=2.83, 95%CI:[0.45, 18.00], I^2^ = 0%, Z=1.10(*P =0.27*); **Figure 5A**), when the risk of hyperthyroidism of grades 3-5 was assessed (14, 18, 39). The corresponding funnel plot was shown in the supplement (**Supplementary Figure 5A**) (14, 18, 39).

Compared with placebo (PD-1/PD-L1 VS. Placebo), the risk of hyperthyroidism of all grades was significantly higher (OR=4.76, 95%CI:[2.17, 10.41], I^2^ = 55%, Z=3.90(*P <*0.0001); **Figure 4B**) (5, 6, 27–29, 33, 35, 45). Through subgroup analysis, high heterogeneity (I^2^ = 55%) was considered to be mainly caused by PD-1 related NSCLC subgroup (I^2^ = 70%, **Figure 4B**) (27, 29). No obvious publication bias was found in the corresponding funnel plot (**Supplementary Figure 4B**). No significant results was noted (OR=3.00, 95%CI:[0.31, 28.89], I^2^ = 0%, Z=0.95 (*P =0.34*); **Figure 5B**), when the risk of hyperthyroidism of grades 3-5 was calculated (28, 29). The corresponding funnel plot was shown in the supplement (**Supplementary Figure 5B**) (28, 29).

When PD-1/PD-L1 combined with chemotherapy was compared with chemotherapy (PD-1/PD-L1+Chemotherapy VS. Chemotherapy), the risk of hyperthyroidism of all grades was found to be significantly higher (OR=4.38, 95%CI:[2.80, 6.85], I^2^ = 0%, Z=6.48(*P <*0.00001); **Figure 4C**) in the PD-1/PD-L1 related group (7, 8, 11, 16, 17, 30, 31). No heterogeneity (I^2^ = 0%) was found (**Figure 4C**). No obvious publication bias was found in the corresponding funnel plot (**Supplementary Figure 4C**). No significant results was noted (OR=3.06, 95%CI:[0.77, 12.10], I^2^ = 0%, Z=1.60(*P =0.11*); **Figure 5C**), when the risk of hyperthyroidism of grades 3-5 was assessed (7, 17, 30, 31). The corresponding funnel plot was shown in the supplement (**Supplementary Figure 5C**) (7, 17, 30, 31).

When PD-1/PD-L1 combined with CTLA-4 was compared with PD-1/PD-L1 (PD-1/PD-L1 VS. PD-1/PD-L1+CTLA-4), the risk of hyperthyroidism of all grades was found to be significantly lower (OR=0.31, 95%CI:[0.19, 0.51], I^2^ = 0%, Z=4.53 (*P <*0.00001); **Figure 4D**) in the PD-1/PD-L1 mono-therapy group (6, 40, 49). No heterogeneity (I^2^ = 0%) was found. No obvious publication bias was found in the funnel plot (**Supplementary Figure 5D**). Similar risk trend could also be seen, when the risk of hyperthyroidism of grades 3-5 was assessed (OR=0.11, 95%CI:[0.01, 0.86], I^2^ = 0%, Z=2.11(*P =0.04*); **Figure 5D**) (6, 50). The corresponding funnel plot was shown in the supplement (**Supplementary Figure 5D**) (6, 50).

When PD-1/PD-L1 combined with chemotherapy was compared with PD-1/PD-L1 (PD-1/PD-L1+Chemotherapy VS. PD-1/PD-L1), no statistical analysis results of hyperthyroidism of all grades was found (OR=1.52, 95%CI:[0.91, 2.51], I^2^ = 0%, Z=1.61(*P =0.011*); **Figure 4E**) (11, 18). No heterogeneity (I^2^ = 0%) was found. No obvious publication bias was found in the funnel plot (**Supplementary Figure 4E**). There were too few data to calculate the risk of hyperthyroidism of grades 3-5 (18).

### Risk of Thyroiditis

Thyroiditis was reported in 17 clinical trials (6, 7, 14, 16, 23, 24, 27–29, 34, 35, 37–39, 41, 42, 47–50), 16 of which were used for the final meta-analysis (6, 7, 14, 16, 24, 27–29, 34, 35, 37–39, 41, 42, 47–50).

**Figure 6:**
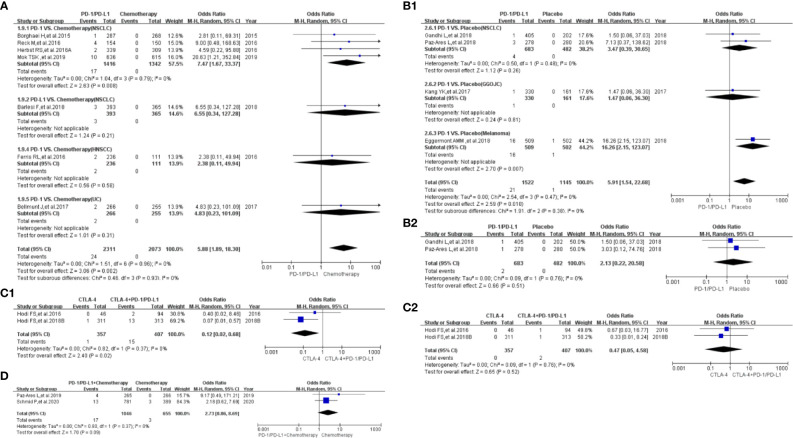
Forest plots of the risk of thyroiditis. **(A)** The risk of all-grade thyroiditis calculated by the random effect (RE) model (PD-1/PD-L1 VS. Chemotherapy): subgroup analysis was conducted based on PD-1/PD-L1 and tumor types in both groups. **(B1)** The risk of all-grade thyroiditis calculated by the random effect (RE) model (PD-1/PD-L1 VS. Placebo): subgroup analysis was conducted based on tumor types in the control group. **(B2)** The risk of thyroiditis for grade 3-5 calculated by the random effect (RE) model (PD-1/PD-L1 VS. Placebo). **(C1)** The risk of all-grade thyroiditis calculated by the random effect (RE) model (CTLA-4 VS. PD-1/PD-L1+CTLA-4): subgroup analysis was conducted based on tumor types in the control group. **(C2)** The risk of thyroiditis for grades 3-5 calculated by the random effect (RE) model (CTLA-4 VS. PD-1/PD-L1+CTLA-4). **(D)** The risk of all-grade thyroiditis calculated by the random effect (RE) model (PD-1/PD-L1+Chemotherapy VS. Chemotherapy).

Compared with chemotherapy (PD-1/PD-L1 VS. Chemotherapy), the risk of thyroiditis of all grades was significantly higher (OR=5.88, 95%CI:[1.89, 18.30], I^2^ = 0%, Z=3.06(*P =0.002*); **Figure 6A**) in PD-1/PD-L1 group (14, 24, 34, 37–39, 42). Subgroup analysis suggested that PD-1 appeared to be associated with a higher incidence risk of thyroiditis in NSCLC subgroup (OR=7.47, 95%CI:[1.67, 33.37], I^2^ = 0%, Z=2.63(*P =0.008*); **Figure 6A**) (14, 37, 39, 42). However, no statistical significant difference was found indifferent subgroups (*P =0.93*, **Figure 6A**). No heterogeneity (I^2^ = 0%) was found (**Figure 6A**). No obvious publication bias was found in the corresponding funnel plot (**Supplementary Figure 6A**). No data of thyroiditis of grades 3-5 was found.

Compared with placebo (PD-1/PD-L1 VS. Placebo), the risk of thyroiditis of all grades was significantly higher (OR=5.91, 95%CI:[1.54, 22.68], I^2^ = 0%, Z=2.59(*P =0.010*); **Figure 6B1**) (27–29, 35). No heterogeneity (I^2 =^ 0%) was found. No obvious publication bias was found in the funnel plot (**Supplementary Figure 6B1**). No statistical significant analysis results was found, when the risk of thyroiditis of grades 3-5 was checked (OR=2.13, 95%CI:[0.22, 20.58], I^2^ = 0%, Z=0.66(*P =0.051*); **Figure 6B2**) (27, 29). The corresponding funnel plot was shown in the supplement (**Supplementary Figure 6B2**) (27, 29).

When PD-1/PD-L1 combined with CTLA-4 was compared with CTLA-4 (CTLA-4 VS. PD-1/PD-L1+CTLA-4), the risk of thyroiditis of all grades was found to be significantly lower (OR=0.12, 95%CI:[0.02, 0.68], I^2^ = 0%, Z=2.40(*P =0.02*); **Figure 6C1**) in CTLA-4 group (41, 49). No heterogeneity (I^2^ = 0%) was found. No obvious publication bias was found in the funnel plot (**Supplementary Figure 6C1**). Similar risk trend could also be found, when the risk of thyroiditis of grades 3-5 was evaluated (OR=0.47, 95%CI:[0.05, 4.58], I^2^ = 0%, Z=0.65(*P =0.52*); **Figure 6C2**) (41, 49). Np heterogeneity (I^2^ = 0%, **Figure 6C2**) was found. The corresponding funnel plot was shown in the supplement (**Supplementary Figure 6C2**) (41, 49).

When PD-1/PD-L1 combined with chemotherapy was compared with chemotherapy (PD-1/PD-L1+Chemotherapy VS. Chemotherapy), no statistical analysis results of thyroiditis of all grades was found (OR=2.73, 95%CI:[0.86, 8.69], I^2^ = 0%, Z=1.70(*P =0.09*); **Figure 6D**) (7, 16). No heterogeneity (I^2^ = 0%) was found. No obvious publication bias was found in the funnel plot (**Supplementary Figure 6D**). No data of thyroiditis of grades 3-5 was found.

## Discussion

Programmed cell death protein 1 (PD-1) and its ligand (PD-L1) inhibitors were developed to overcome the immune escape mechanisms of cancer progression and manipulate the immune system to recognize and attack cancer cells (1). A large number of PD-1/PD-L1 related immune-related toxicities, including thyroid dysfunction, had been reported (1, 4–50), which might be related to this immune regulation mechanism. Clinical manifestations of thyroid dysfunction ranged from life threatening to no signs or symptoms (64–66). Therefore, systematic assessment of the risk of thyroid dysfunction had an important guiding significance for clinical work (1).

Consistent with previous reports (1), hypothyroidism was much more common with PD-1/PD-L1 inhibitors than others (**Table 1**) (4–50). Through comprehensive analysis, we found that the risk of hypothyroidism of all grades in the PD-1/PD-L1 mono-therapy group was significantly higher compared to the chemotherapy arm (**Figure 2A**) (4, 11, 12, 14, 15, 18, 19, 24–26, 32, 34, 37–39, 42–44). Similar results could also be noted, when the control group was placebo or CTLA-4 (**Figures 2B, E**) (5, 6, 27–29, 33, 35, 36, 46, 49). When PD-1/PD-L1 was combined with other treatments for cancer patients, the risk of hypothyroidism of all grades was also significantly increased (**Figures 2C, D, F**) (6–11, 16, 17, 30–32, 40, 49). Subgroup analysis suggested that PD-1 appeared to be associated with a higher incidence risk of hypothyroidism compared to PD-L1 (**Supplementary Figure 7**) (4, 14, 15, 18, 19, 25, 32, 34, 37–39, 42–44). But this difference between PD-1 and PD-L1 subgroup was not statistical significant (**Supplementary Figure 7**) (4, 14, 15, 18, 19, 25, 32, 34, 37–39, 42–44). Due to the lack of clinical trials on PD-1 and PD-L1 head-to-head comparisons, we could not clarify the difference in the risk of hypothyroidism between the two. For the existence of heterogeneity (**Figures 2A–C, E**), we conducted a sufficient stratified subgroup analysis and inferred the source of the heterogeneity. Furthermore, no obvious publication bias was found among all the enrolled clinical trials (**Supplementary Figure 2**). Therefore, the conclusion that PD-1/PD-L1 increased the risk of hypothyroidism of all grades was considered to be much more reliable. No significant results was noted, when the risk of hypothyroidism of grades 3-5 was calculated (**Figure 3** and **Supplementary Figure 3**).

Drug-induced thyroid dysfunction is one of the common causes of hyperthyroidism (67). Whether PD-1/PD-L1 inhibitors were used alone or in combination with other drugs, it indicated that PD-1/PD-L1 inhibitors increased the risk of hyperthyroidism of all grades (**Figures 4A–D**). When PD-1/PD-L1 combined with chemotherapy was compared with PD-1/PD-L1, no statistical analysis results of hyperthyroidism of all grades was found (**Figure 4E**) (11, 18). Through the above analysis, we clarified the role of PD-1/PD-L1 inhibitors in increasing the risk of hyperthyroidism of all grades (**Figure 4** and **Supplementary Figure 4**) (5–12, 14–18, 24–31, 33–35, 37–40, 42, 43, 45–50). Through subgroup analysis, high heterogeneity (I^2 =^ 55%) was considered to be mainly caused by PD-1 related NSCLC subgroup (I^2 =^ 70%, **Figure 4B**) (27, 29). No obvious publication bias was found among all the enrolled clinical trials (**Supplementary Figure 4**). Though similar incidence trend could also be seen in the assessment of hypothyroidism of grades 3-5 (**Figure 5**), statistical significant result was only found in (**Figure 5D**). Since only two clinical trials were included (**Figure 5D**), the analysis results need to be further verified.

In the clinical trials included in the study, the incidence rate of thyroiditis was lower than those of hyperthyroidism and hypothyroidism (**Table 1**). Similar to the previous analysis results, PD-1/PD-L1 inhibitors played the same role in increasing the risk of thyroiditis (**Figure 6**). No obvious heterogeneity and publication bias was found among all enrolled clinical trials (**Figure 6** and **Supplementary Figure 6**) (6, 7, 14, 16, 24, 27–29, 34, 35, 37–39, 41, 42, 47–50).

Thyroid dysfunction had also been reported in other 5 PD-1/PD-L1 investigated clinical trials (13, 20–23). For the heterogeneity among these 5 clinical trials, it was impossible for us to conduct a meta-analysis. However, we found that sunitinib might play a similar role to PD-1/PD-L1 on increasing the risk of thyroid dysfunction (21–23).

By reviewing and analyzing PD-1/PD-L1 related literature (4–50), we found that PD-1/PD-L1 increased the risk of thyroid dysfunction. It reminds us that we need to monitor and evaluate the thyroid function status in time for patients receiving PD-1/PD-L1 treatment to prevent the occurrence of adverse events (1–3, 64–67).

### Strengths and Limitations

Strengths: This meta-analysis was conducted according to the PRISMA guidelines. The literature searching process was put into practice in accordance with the PICOS principle. The quality of all enrolled clinical trials was high. Stratification and subgroup analyses were conducted as much as possible. Therefore, the conclusion was much more reliable.

Limitations: First, some clinical trials related to PD-1/PD-L1 inhibitors cannot be included for meta-analysis due to obvious heterogeneity. Second, the low number of studies that reported the data of thyroid dysfunction made it difficult to get a definite conclusion.

## Conclusion

Whether used alone or in combination with other anti-tumor drugs, PD-1/PD-L1 inhibitors increased the risk of thyroid dysfunction, especially for hypothyroidism. Furthermore, PD-1/PD-L1 was better than chemotherapy and CTLA-4 in increasing the risk of thyroid dysfunction.

## Data Availability Statement

The original contributions presented in the study are included in the article/**Supplementary Material**. Further inquiries can be directed to the corresponding authors.

## Author Contributions

The corresponding authors (YPS and GS) had the right to deal with all the data and were responsible for the decision to submit this manuscript for publication. YT, RL, YL, ML, YXS, YZ, AG and QW had the full data of the manuscript. YT, RL, YL, ML, and YXS were responsible for checking and evaluating the quality of the data and enrolled studies. YT was appointed for writing the draft of this manuscript. All authors contributed to the article and approved the submitted version.

## Funding

This study was funded by the Academic Promotion Program of Shandong First Medical University (2019QL025; YPS), Natural Science Foundation of Shandong Province (ZR2019MH042; YPS), Jinan Science and Technology Program (201805064; YPS), the National Science and Technology Major Project of the Ministry of Science and Technology of China (2020ZX09201025; GS), Postdoctoral Innovation Project of Jinan (YT), the National Natural Science Foundation of China (No. 81170087; GS), the Provincial Natural Science Foundation of Shandong (ZR2018MH003; GS), the Clinical Medical Science and Technology Innovation Program of Jinan (201805004; GS).

## Conflict of Interest

The authors declare that the research was conducted in the absence of any commercial or financial relationships that could be construed as a potential conflict of interest.

## Supplementary Material

Supplementary Figure 1 A summary table of review authors’ judgements for each risk of bias item for each study.

Supplementary Figure 2 Funnel plots of the risk of all-grade hypothyroidism. **(A)** The risk of hypothyroidism calculated by the random effect (RE) model (PD-1/PD-L1 VS. Chemotherapy): subgroup analysis was conducted based on PD-1/PD-L1, chemotherapy drugs and tumor types in both groups. **(B)** The risk of hypothyroidism calculated by the random effect (RE) model (PD-1/PD-L1 VS. Placebo): subgroup analysis was conducted based on PD-1/PD-L1 and tumor types in both groups. **(C)** The risk of hypothyroidism calculated by the random effect (RE) model (PD-1/PD-L1+Chemotherapy VS. Chemotherapy): subgroup analysis was conducted based on PD-1/PD-L1 and tumor types in both groups. **(D)** The risk of hypothyroidism calculated by the random effect (RE) model (PD-1/PD-L1 VS. PD-1/PD-L1+CTLA-4): subgroup analysis was conducted based on tumor types in the control group. **(E)** The risk of hypothyroidism calculated by the random effect (RE) model (PD-1 VS. CTLA-4): subgroup analysis was conducted based on the PD-1 group. **(F)** The risk of hypothyroidism calculated by the random effect (RE) model (PD-1/PD-L1+Targeted VS. Targeted).

Supplementary Figure 3 Funnel plots of the risk of hypothyroidism for grades 3-5. **(A)** The risk of hypothyroidism calculated by the random effect (RE) model (PD-1/PD-L1 VS. Chemotherapy): subgroup analysis was conducted based on PD-1/PD-L1 and tumor types in both groups. **(B)** The risk of hypothyroidism calculated by the random effect (RE) model (PD-1/PD-L1 VS. Placebo): subgroup analysis was conducted based on PD-1/PD-L1 and tumor types in both groups. **(C)** The risk of hypothyroidism calculated by the random effect (RE) model (PD-1/PD-L1+Chemotherapy VS. Chemotherapy): subgroup analysis was conducted based on PD-1/PD-L1 and tumor types in both groups.

Supplementary Figure 4 Funnel plots of the risk of all-grade hyperthyroidism. **(A)** The risk of hyperthyroidism calculated by the random effect (RE) model (PD-1/PD-L1 VS. Chemotherapy): subgroup analysis was conducted based on PD-1/PD-L1 and tumor types in both groups. **(B)** The risk of hyperthyroidism calculated by the random effect (RE) model (PD-1/PD-L1 VS. Placebo): subgroup analysis was conducted based on PD-1/PD-L1 and tumor types in both groups. **(C)** The risk of hyperthyroidism calculated by the random effect (RE) model (PD-1/PD-L1+Chemotherapy VS. Chemotherapy): subgroup analysis was conducted based on PD-1/PD-L1 and tumor types in both groups. **(D)** The risk of hyperthyroidism calculated by the random effect (RE) model (PD-1/PD-L1 VS. PD-1/PD-L1+CTLA-4): subgroup analysis was conducted based on tumor types in the control group. **(E)** The risk of hyperthyroidism calculated by the random effect (RE) model (PD-1/PD-L1+chemotherapy VS. PD-1/PD-L1): subgroup analysis was conducted based on PD-1/PD-L1 in both groups.

Supplementary Figure 5 Funnel plots of the risk of hyperthyroidism for grades 3-5. **(A)** The risk of hyperthyroidism calculated by the random effect (RE) model (PD-1/PD-L1 VS. Chemotherapy). **(B)** The risk of hyperthyroidism calculated by the random effect (RE) model (PD-1/PD-L1 VS. Placebo). **(C)** The risk of hyperthyroidism calculated by the random effect (RE) model (PD-1/PD-L1+Chemotherapy VS. Chemotherapy): subgroup analysis was conducted based on PD-1/PD-L1 and tumor types in both groups. **(D)** The risk of hyperthyroidism calculated by the random effect (RE) model (PD-1/PD-L1 VS. PD-1/PD-L1+CTLA-4).

Supplementary Figure 6 Funnel plots of the risk of thyroiditis. **(A)** The risk of all-grade thyroiditis calculated by the random effect (RE) model (PD-1/PD-L1 VS. Chemotherapy): subgroup analysis was conducted based on PD-1/PD-L1 and tumor types in both groups. **(B1)** The risk of all-grade thyroiditis calculated by the random effect (RE) model (PD-1/PD-L1 VS. Placebo): subgroup analysis was conducted based on tumor types in the control group. **(B2)** The risk of thyroiditis for grade 3-5 calculated by the random effect (RE) model (PD-1/PD-L1 VS. Placebo). **(C1)** The risk of all-grade thyroiditis calculated by the random effect (RE) model (CTLA-4 VS. PD-1/PD-L1+CTLA-4): subgroup analysis was conducted based on tumor types in the control group. **(C2)** The risk of thyroiditis for grades 3-5 calculated by the random effect (RE) model (CTLA-4 VS. PD-1/PD-L1+CTLA-4). **(D)** The risk of all-grade thyroiditis calculated by the random effect (RE) model (PD-1/PD-L1+Chemotherapy VS. Chemotherapy).

Supplementary Figure 7 Forest plots of the risk of all-grade hypothyroidism. The risk of hypothyroidism calculated by the random effect (RE) model (PD-1/PD-L1 VS. Chemotherapy): subgroup analysis was conducted based on PD-1/PD-L1 in both groups.

Supplementary Figure 8 Forest plots of the risk of all-grade hyperthyroidism. The risk of hyperthyroidism calculated by the random effect (RE) model (PD-1/PD-L1 VS. Chemotherapy): subgroup analysis was conducted based on PD-1/PD-L1 in both groups.

## Abbreviations

PD-1, Programmed Cell Death-1; PD-L1, Programmed Cell Death Ligand 1; PRISMA, Preferred Reporting Items for Systematic Reviews and Meta-Analyses; PICOS, Participants, Interventions, Comparisons, Outcomes, and Study design; N/A, No Available; HR, Hazard Ratios; OR, Odds Ratio; CI, Confidence Interval; RE, Random Effect; NSCLC, Non-Small Cell Lung Cancer; SCLC, Small Cell Lung Cancer; OSCC, Oesophageal Squamous Cell Carcinoma; HNSCC, Head and Neck Squamous Cell Carcinoma; UC, Urothelial Cancer; BC, Breast Cancer; RCC, Renal Cell Carcinoma; NOS, Newcastle-Ottawa scale; TNBC, Triple-Negative Breast Cancer; GGOJC, Gastric or Gastro-Oesophageal Junction Cancer.
