# Supplementary figures and images for: The Risk of Immune-Related Thyroid Dysfunction Induced by PD-1/PD-L1 Inhibitors in Cancer Patients: An Updated Systematic Review and Meta-Analysis
Source: Front Oncol. 2021 Jul 12;11:667650. doi: 10.3389/fonc.2021.667650 (PMC8312489; doi:10.3389/fonc.2021.667650)

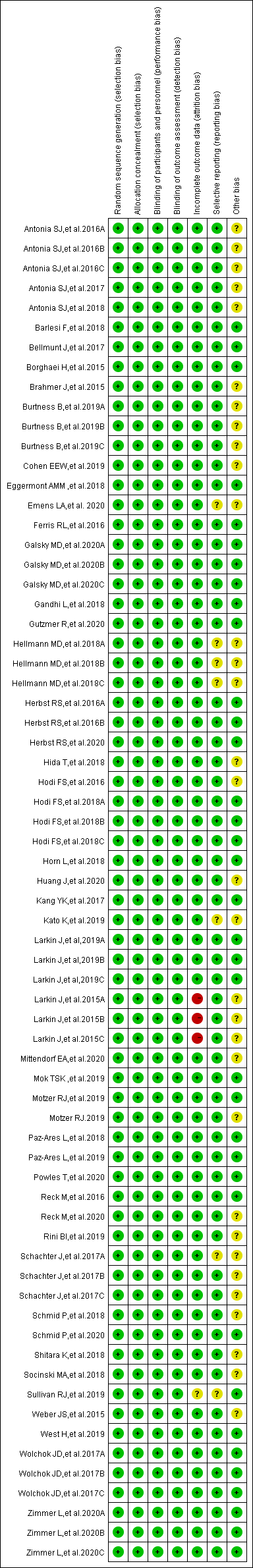

Supplement: Supplementary Figure 1 — A summary table of review authors’ judgements for each risk of bias item for each study. [file Image_1.tif]

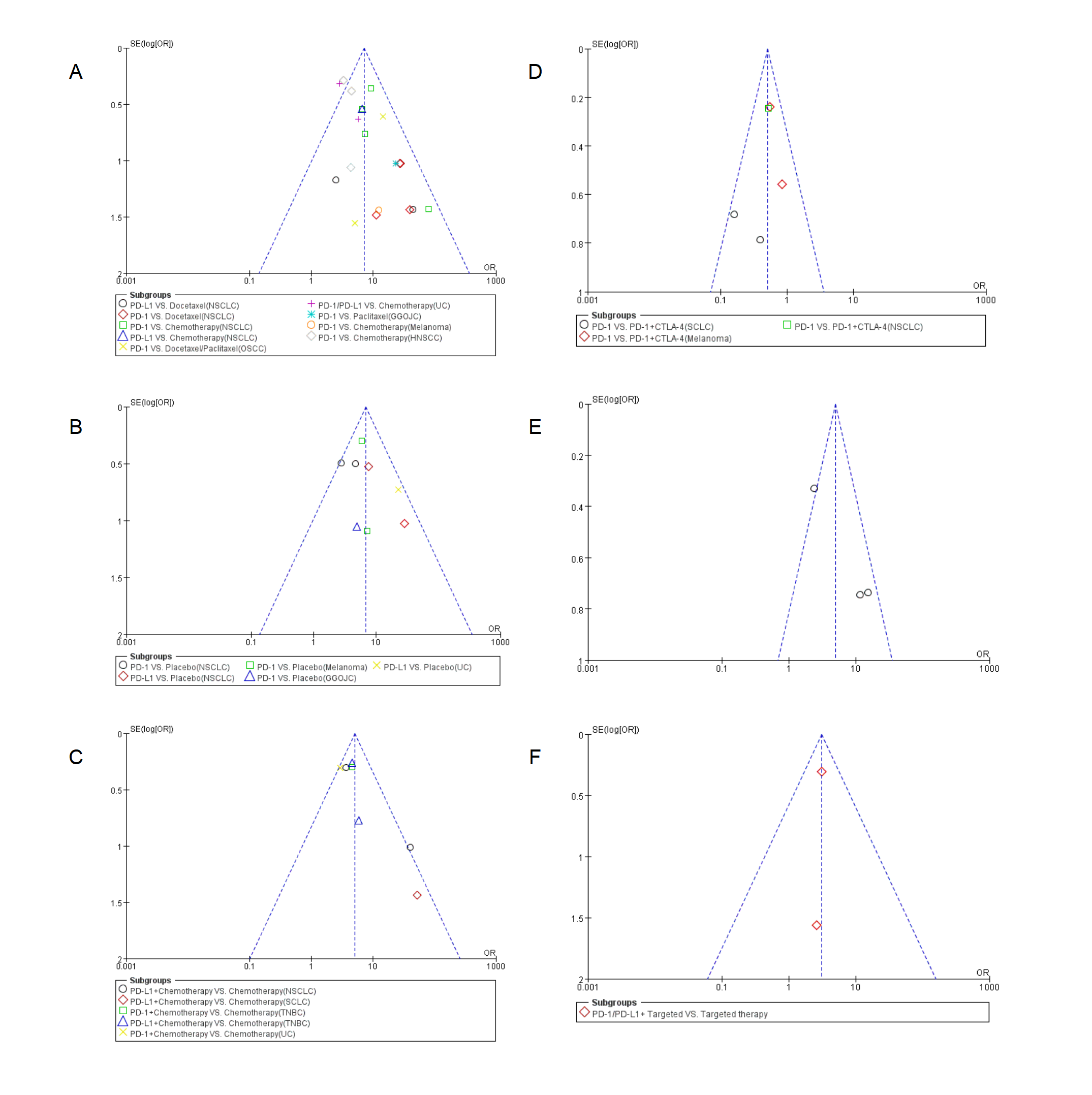

Supplement: Supplementary Figure 2 — Funnel plots of the risk of all-grade hypothyroidism. (A) The risk of hypothyroidism calculated by the random effect (RE) model (PD-1/PD-L1 VS. Chemotherapy): subgroup analysis was conducted based on PD-1/PD-L1, chemotherapy drugs and tumor types in both groups. (B) The risk of hypothyroidism calculated by the random effect (RE) model (PD-1/PD-L1 VS. Placebo): subgroup analysis was conducted based on PD-1/PD-L1 and tumor types in both groups. (C) The risk of hypothyroidism calculated by the random effect (RE) model (PD-1/PD-L1+Chemotherapy VS. Chemotherapy): subgroup analysis was conducted based on PD-1/PD-L1 and tumor types in both groups. (D) The risk of hypothyroidism calculated by the random effect (RE) model (PD-1/PD-L1 VS. PD-1/PD-L1+CTLA-4): subgroup analysis was conducted based on tumor types in the control group. (E) The risk of hypothyroidism calculated by the random effect (RE) model (PD-1 VS. CTLA-4): subgroup analysis was conducted based on the PD-1 group. (F) The risk of hypothyroidism calculated by the random effect (RE) model (PD-1/PD-L1+Targeted VS. Targeted). [file Image_2.tif]

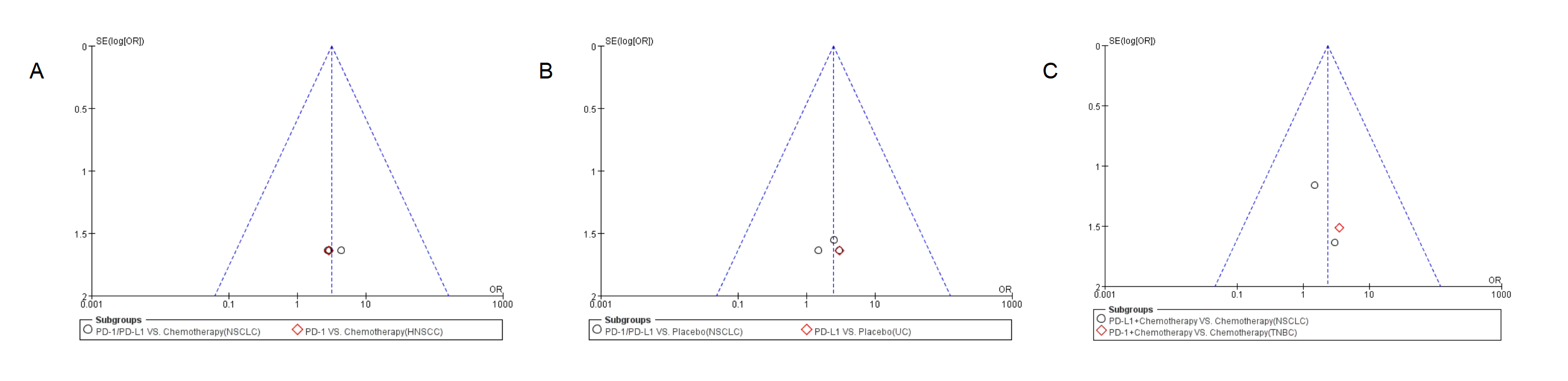

Supplement: Supplementary Figure 3 — Funnel plots of the risk of hypothyroidism for grades 3-5. (A) The risk of hypothyroidism calculated by the random effect (RE) model (PD-1/PD-L1 VS. Chemotherapy): subgroup analysis was conducted based on PD-1/PD-L1 and tumor types in both groups. (B) The risk of hypothyroidism calculated by the random effect (RE) model (PD-1/PD-L1 VS. Placebo): subgroup analysis was conducted based on PD-1/PD-L1 and tumor types in both groups. (C) The risk of hypothyroidism calculated by the random effect (RE) model (PD-1/PD-L1+Chemotherapy VS. Chemotherapy): subgroup analysis was conducted based on PD-1/PD-L1 and tumor types in both groups. [file Image_3.tif]

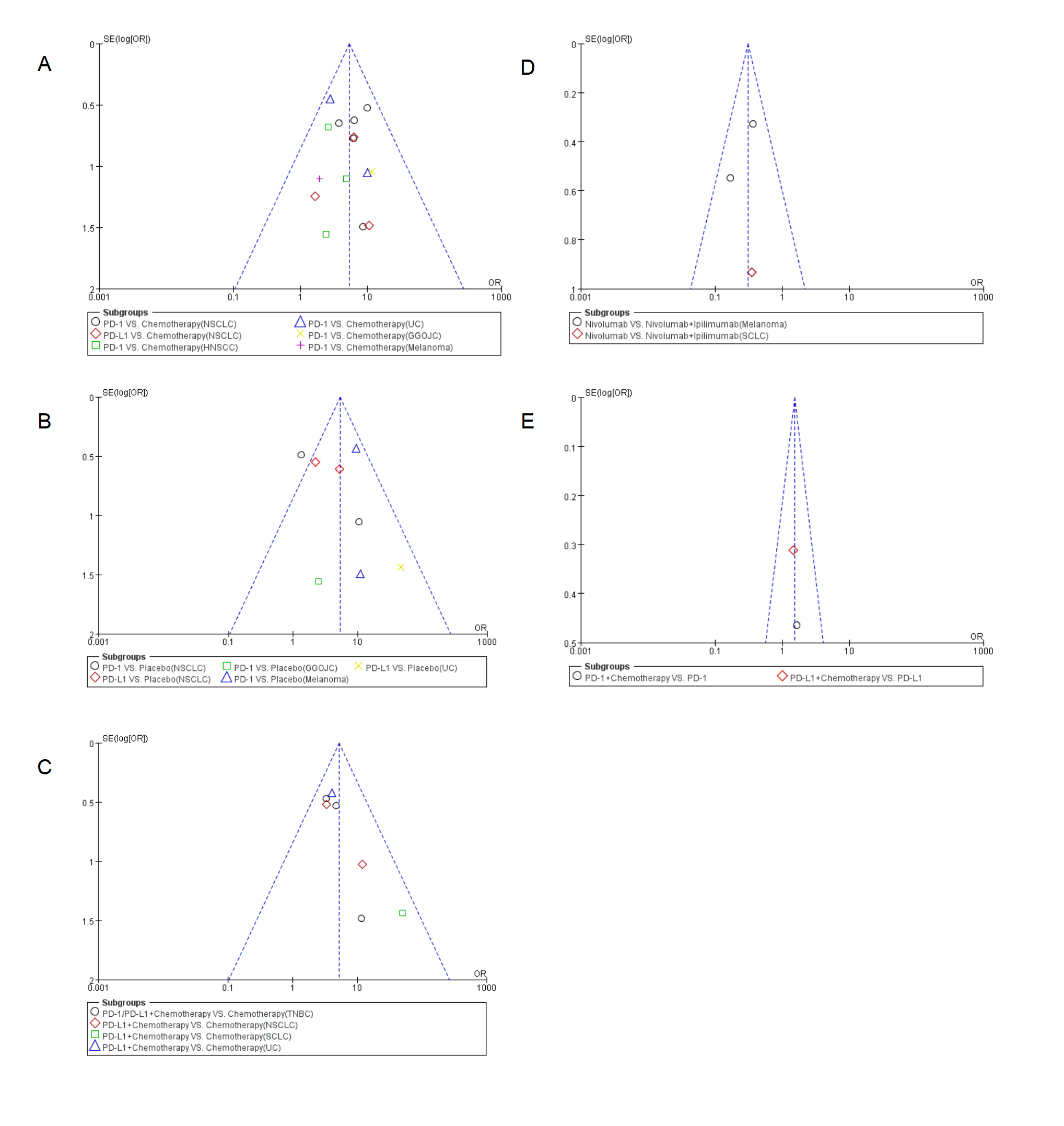

Supplement: Supplementary Figure 4 — Funnel plots of the risk of all-grade hyperthyroidism. (A) The risk of hyperthyroidism calculated by the random effect (RE) model (PD-1/PD-L1 VS. Chemotherapy): subgroup analysis was conducted based on PD-1/PD-L1 and tumor types in both groups. (B) The risk of hyperthyroidism calculated by the random effect (RE) model (PD-1/PD-L1 VS. Placebo): subgroup analysis was conducted based on PD-1/PD-L1 and tumor types in both groups. (C) The risk of hyperthyroidism calculated by the random effect (RE) model (PD-1/PD-L1+Chemotherapy VS. Chemotherapy): subgroup analysis was conducted based on PD-1/PD-L1 and tumor types in both groups. (D) The risk of hyperthyroidism calculated by the random effect (RE) model (PD-1/PD-L1 VS. PD-1/PD-L1+CTLA-4): subgroup analysis was conducted based on tumor types in the control group. (E) The risk of hyperthyroidism calculated by the random effect (RE) model (PD-1/PD-L1+chemotherapy VS. PD-1/PD-L1): subgroup analysis was conducted based on PD-1/PD-L1 in both groups. [file Image_4.tif]

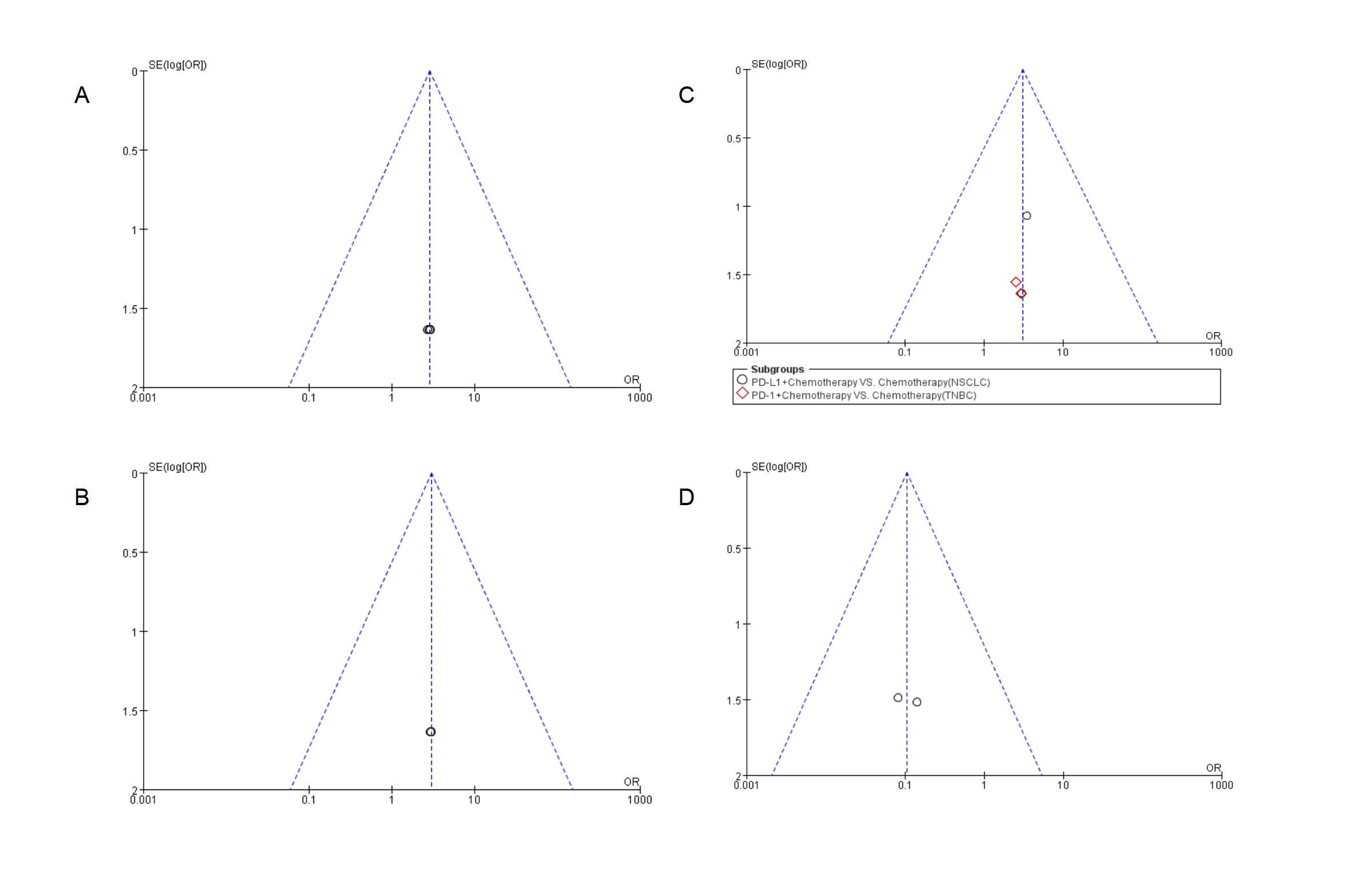

Supplement: Supplementary Figure 5 — Funnel plots of the risk of hyperthyroidism for grades 3-5. (A) The risk of hyperthyroidism calculated by the random effect (RE) model (PD-1/PD-L1 VS. Chemotherapy). (B) The risk of hyperthyroidism calculated by the random effect (RE) model (PD-1/PD-L1 VS. Placebo). (C) The risk of hyperthyroidism calculated by the random effect (RE) model (PD-1/PD-L1+Chemotherapy VS. Chemotherapy): subgroup analysis was conducted based on PD-1/PD-L1 and tumor types in both groups. (D) The risk of hyperthyroidism calculated by the random effect (RE) model (PD-1/PD-L1 VS. PD-1/PD-L1+CTLA-4). [file Image_5.tif]

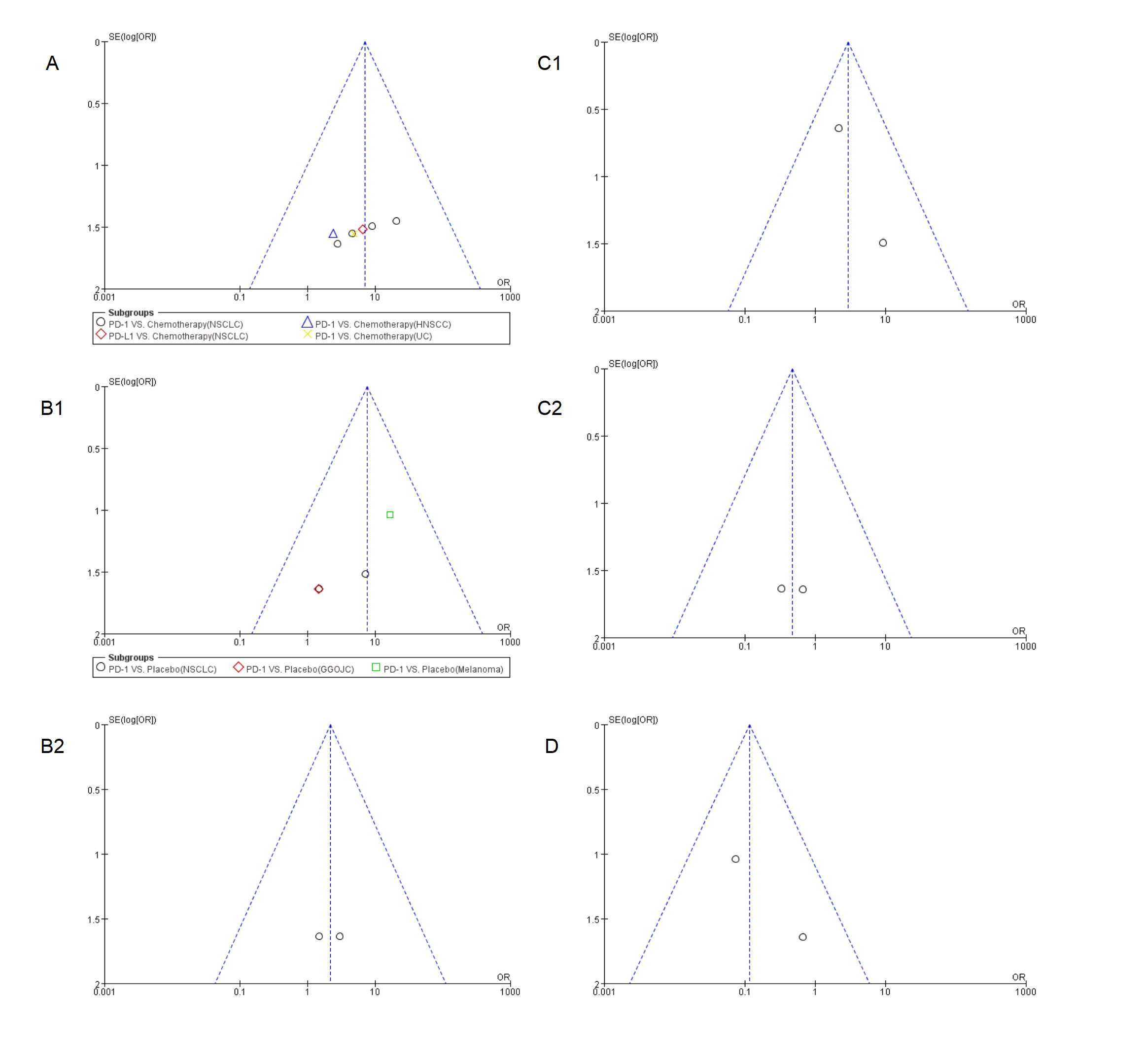

Supplement: Supplementary Figure 6 — Funnel plots of the risk of thyroiditis. (A) The risk of all-grade thyroiditis calculated by the random effect (RE) model (PD-1/PD-L1 VS. Chemotherapy): subgroup analysis was conducted based on PD-1/PD-L1 and tumor types in both groups. (B1) The risk of all-grade thyroiditis calculated by the random effect (RE) model (PD-1/PD-L1 VS. Placebo): subgroup analysis was conducted based on tumor types in the control group. (B2) The risk of thyroiditis for grade 3-5 calculated by the random effect (RE) model (PD-1/PD-L1 VS. Placebo). (C1) The risk of all-grade thyroiditis calculated by the random effect (RE) model (CTLA-4 VS. PD-1/PD-L1+CTLA-4): subgroup analysis was conducted based on tumor types in the control group. (C2) The risk of thyroiditis for grades 3-5 calculated by the random effect (RE) model (CTLA-4 VS. PD-1/PD-L1+CTLA-4). (D) The risk of all-grade thyroiditis calculated by the random effect (RE) model (PD-1/PD-L1+Chemotherapy VS. Chemotherapy). [file Image_6.tif]

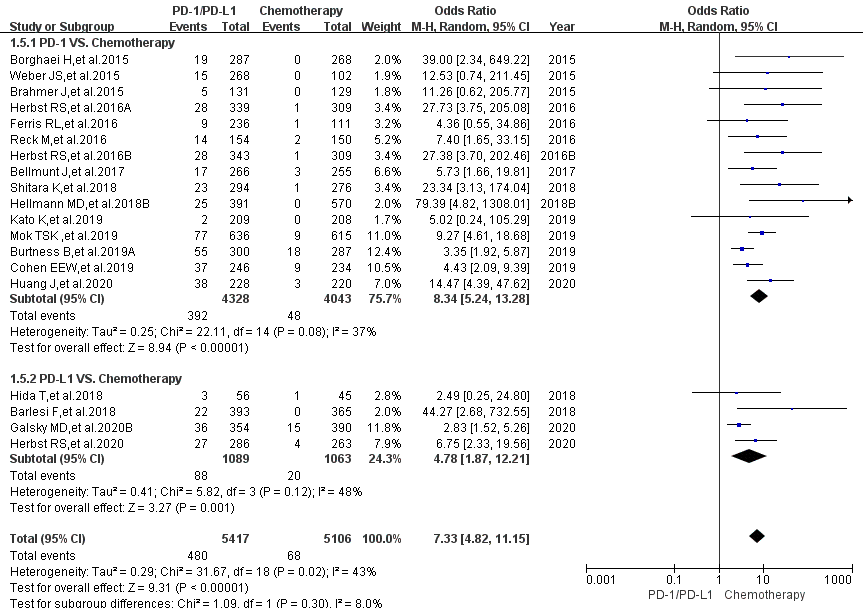

Supplement: Supplementary Figure 7 — Forest plots of the risk of all-grade hypothyroidism. The risk of hypothyroidism calculated by the random effect (RE) model (PD-1/PD-L1 VS. Chemotherapy): subgroup analysis was conducted based on PD-1/PD-L1 in both groups. [file Image_7.tif]

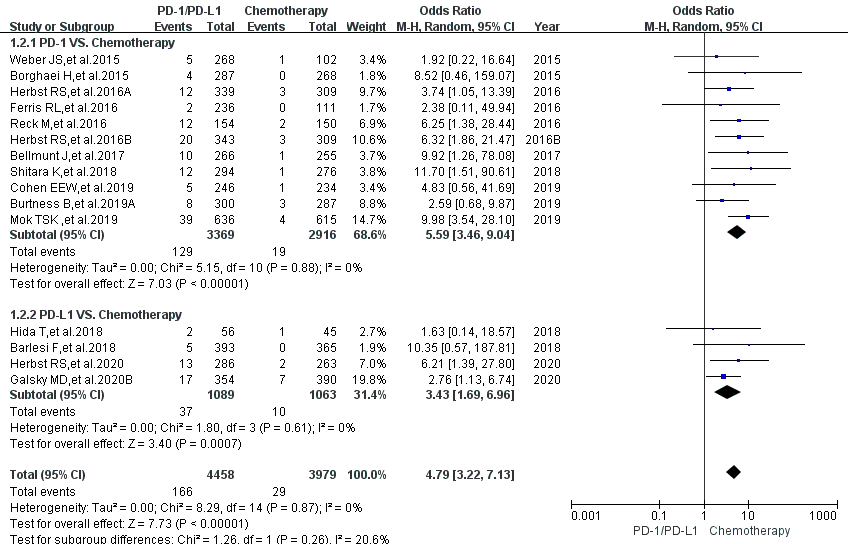

Supplement: Supplementary Figure 8 — Forest plots of the risk of all-grade hyperthyroidism. The risk of hyperthyroidism calculated by the random effect (RE) model (PD-1/PD-L1 VS. Chemotherapy): subgroup analysis was conducted based on PD-1/PD-L1 in both groups. [file Image_8.tif]
